# The Uses of Hypnotism in Dentistry

**Published:** 1893-05

**Authors:** Thomas Fillebrown


### THE USES OF HYPNOTISM IN DENTISTRY.[1]

BY THOMAS FILLEBROWN, M.D., D.M.D.

Mr. President and Gentlemen,—I am happy to answer your invitation to be present this evening and say a word about the use of hypnotic suggestion in dentistry, and to demonstrate the manner of its application. Dr. Osgood has reviewed the history and de-

[1] "Smoke-talk" at a meeting of Harvard Odontological Society, held October 27, 1892.

scribed the nature of hypnotism and the uses of suggestion as a therapeutic agent; I will therefore say nothing on those points.

I am aware that for many years hypnotic anæsthesia has been successfully applied for dental operations to exceptional patients that could be hypnotized very deeply.

I remember my father did dentistry for a mesmerized patient previous to 1850. The patient had no knowledge of what was done, and suffered no pain. Others have done the same thing. Few patients reach this deep sleep, and it is of little importance in our specialty.

To make the lesser degree of hypnotic sleep useful and available is the problem I attempted to solve, and succeeded.

Professor Bernheim, Dr. Moll, and all other writers on hypnotism, state that pain will rouse the patient from light sleep, and they therefore cannot endure any prolonged operation.

Previous to my successful application, about one year ago, I am not aware that any one had made suggestion during the light degree of hypnotic sleep effective as a dental obtundent.

For twenty-five years I had considered this subject and believed there was something of value in it, but could not understand how it could be applied so as to be of use until I read Dr. Osgood's account of the case which he has referred to this evening, in which he said, "The suggestion ought to have been repeated in order to have kept her asleep until the culmination of labor." Here light dawned upon me, and I said, "If I can repeat the suggestion often enough, I can keep the patient in the anæsthetic condition during a prolonged operation."

I immediately acted upon this idea and accomplished the object, and maintained anæsthesia of sensitive dentine when the hypnotic sleep was so light that patients would declare they had not been hypnotized at all.

I hypnotize the patient before I begin, then touch the tooth to be operated on with my finger, and say, "This tooth and gum are anæsthetized, the sensitiveness has gone out of it, the silk will not hurt the gum. The cutting of the tooth will not hurt you, the dentine is anæsthetized. If it hurts a little, you will not mind it."

If the patient flinches, I wait a moment and renew my suggestion, and then again cut while keeping up the suggestion, talking all the while, the suggestion offsetting the cutting until I have accomplished the work without causing any pain to the patient.

Another great benefit which obtains is, the fears and nervousness of the patient are removed, so that, instead of grasping my hands and jumping at my motions, they rest entirely quiet until actual trouble comes, and then so soon as it ceases their resistance stops.

Patients get up from an hour's sitting rested and refreshed. This is not the experience of a dental patient ordinarily. The practice of hypnotism has also remarkably increased my own power to manage and quiet patients and have things go well without any attempt to hypnotize.

With me the practice of hypnotism and the application of it to our calling is past the stage of experiment. At first I urged it upon patients; now I leave them quite free to choose. Most of my patients now know of it, and are ready and glad to have it administered, in order to save suffering.

I find it undesirable to keep a patient hypnotized long without waking, as it tires them. Some have roused up a little and said, "Wake me up, I am tired." It has become my habit to suggest rest and comfort, and wake the patient, and let them remain awake for a while, until the anæsthesia is needed again.

I have also noticed that after persons have been hypnotized a few times they get into the condition that needs hypnosis very much less than at first. They remain quiet, and the same sensitiveness does not appear in the teeth, and they appear to be decidedly different persons from what they were before.

An analysis of cases described in a paper I read before the Massachusetts Dental Society, on "Hypnotism applied to Dentistry," showed that in forty per cent. the anæsthesia of the dentine was complete, in thirty-five per cent. sufficient for all practical purposes, and in twenty-five per cent. additional means had to be applied. My later experience bears out this favorable showing.

I will describe a few of my most recent cases.

*October* 22.—A young girl, fifteen years old, had been a patient of mine from a child. I had never been able to do more than fill her teeth with cement in an indifferent way, with the cavity half excavated and with no attempt at making any proper shape. She was utterly unable to cease or abate her active resistance, though she would have been glad to do so.

I hypnotized her last spring, and she went through a whole sitting without touching my hand at all, a thing before unknown. She is now still more tractable than ever, and I can find no fault with her. At her last sitting I prepared an old cavity and filled it with gold, using excavator, chisel, burr, and plugger just as I wished. She sat and allowed all this without any resistance at all. I am sure of a desirable sitting to-morrow.

On the 21st of this month a lady wished to have a tooth extracted, but did not wish to inhale any anæsthetic. I said, "Try hypnotism." After explanation she consented. She hypnotized to a light degree; her resistance was all gone, and she felt perfectly willing I should proceed. She said she felt it a little, but did not suffer at all.

On the 19th I used hypnotism for a patient who had always been very nervous, and had great success in quieting her and almost entirely relieving the sensibility of the dentine.

On September 17 and 19, and October 11, 17, and 19 I gave sittings to a lady who had always been a terror to herself and dentist. She was very nervous and considerably rheumatic, and had been unable to think of having anything done to her teeth for two years. At the first sitting there was extreme nervousness and tremor, rapid pulse, not less than 100, and muscular system in a state of spasm. She was too excited to be hypnotized at once. I used wakeful suggestion, and found it effective. I talked to her quietly and seriously, and assured success. In a few minutes her pulse was about normal, and she said she felt entirely rested. I then hypnotized her, and thoroughly prepared for filling two proximal cavities in her front teeth. As she had just gotten up from a sick-bed, I thought best to let this suffice for the first time.

After every subsequent visit she left my chair feeling in a better condition than when she sat down. Her sittings were most of them in the afternoon, because she always felt better at that time.

Once she wished to come in the morning to see if it would not help her while feeling poorly. It fulfilled our expectations; she felt very much better than on other mornings.

This case shows how difficult it is to separate a specialty from the general knowledge and practice of medicine.

I agree with Dr. Osgood that whatever degree a man has, he must have considerable medical knowledge to be able to properly use this wonderful agent of suggestion either in the wakeful or in the hypnotic condition.

On the 15th I had a case in which I met the phenomenon of laughing, of which Dr. Osgood has spoken. More than half the time for hypnotizing had passed when she burst out laughing, saying, "You can't hypnotize me: I don't feel sleepy a particle."

I replied, "I think you are mistaken. I guess we will keep on." In less than fifteen seconds after that she was profoundly hypnotized.

I suggested absence of pain or fear. She opened her mouth readily. I extracted a tooth, giving her no shock whatever and no pain.

It is only in exceptional cases that hypnotic anæsthesia is sufficient for extraction.

On the 12th I had another case for filling, which was entirely successful. This patient had previously had some mental treatment for disease.

I find such patients more susceptible to hypnotism than are those who have had nothing of the kind.

*September* 14.—I did some dentistry for a young boy for whom I operated last spring, and at that time he was very difficult to manage. While he was then willing to be hypnotized, his mother, who was with him, was not quite reconciled, and I could not use it.

On the 17th instant he had more to be done, and said, "They have all been converted out to the house, and you may hypnotize me." I did so, and it worked admirably. I could cut the dentine, and cleanse and form the cavities well, and fill them satisfactorily to myself without any resistance from him whatever. I was interested, when he woke up, in his rather graphic description of his condition. He said, "I felt just the same as a fellow does when he wakes up in the morning and finds his body all asleep."

These cases are sufficient to show my progress in the use of hypnotism since my published list, and I will not take more time now, but proceed to the more practical and useful part, the application of the method.

[Dr. Fillebrown here tried seven subjects, neither of which had ever been hypnotized, and succeeded in each case in obtaining a pronounced hypnotic sleep.]

